# Draft Genome Sequences of Six Multidrug-Resistant Clinical Strains of Acinetobacter baumannii, Isolated at Two Major Hospitals in Kuwait

**DOI:** 10.1128/genomeA.00264-18

**Published:** 2018-04-19

**Authors:** Kother Nasser, Abu Salim Mustafa, Mohd Wasif Khan, Prashant Purohit, Inaam Al-Obaid, Rita Dhar, Wadha Al-Fouzan

**Affiliations:** a Department of Microbiology, Faculty of Medicine, Kuwait University, Kuwait City, Kuwait; b OMICS Research Unit, Research Core Facility, Health Sciences Centre, Kuwait University, Kuwait City, Kuwait; c Department of Medical Microbiology, Al-Sabah Hospital, Kuwait City, Kuwait; d Department of Medical Microbiology, Farwaniya Hospital, Kuwait City, Kuwait

## Abstract

Acinetobacter baumannii is an important opportunistic pathogen in global health care settings. Its dissemination and multidrug resistance pose an issue with treatment and outbreak control. Here, we present draft genome assemblies of six multidrug-resistant clinical strains of A. baumannii isolated from patients admitted to one of two major hospitals in Kuwait.

## GENOME ANNOUNCEMENT

Acinetobacter baumannii has steadily raised concerns in the medical field over the past decade as one of the most dangerous multidrug-resistant (MDR) human pathogens in health care settings and conflict zones (1). This opportunistic Gram-negative organism is the most pathogenic of the four genotypically related species in the Acinetobacter calcoaceticus*-* Acinetobacter baumannii (ACB) complex (2). Identification and genotyping of A. baumannii isolates are important for contact tracing and epidemiologic surveillance in outbreak conditions (3).

Whole-genome sequencing, by using next-generation DNA sequencing technologies, offers an increasingly attainable new path of bacterial investigation and outbreak control, through which partial or complete genomes can be examined based on nucleotide differences at a single site (single nucleotide polymorphisms [SNPs]) across the entire genome (4). In order to identify genetic variations in strains from Kuwait, we performed whole-genome sequencing of six multidrug-resistant A. baumannii strains isolated from patients admitted to one of two major hospitals in Kuwait. The sequence data were analyzed to determine the number of SNPs in the genomes.

Six clinical isolates of A. baumannii, three isolates each from Al-Sabah Hospital (KUSSH13, KUSSH35, and KUSSH36) and Farwaniya Hospital (KUFAR40, KUFAR42, and KUFAR44) (Table 1), were grown on blood agar plates, and single colonies were suspended in nuclease-free water. The bacterial suspensions were heated at 95°C for 25 min, and DNA was purified using the QIAamp DNA minikit (Qiagen, Hilden, Germany) and quantified using the Qubit double-stranded DNA (dsDNA) broad-range assay (Thermo Fisher Scientific). DNA libraries were prepared using the Nextera XT DNA library preparation kit (Illumina, San Diego, CA). The genomic DNA was sequenced using Illumina MiSeq paired-end (2 × 150-bp) sequencing technology (5). Output reads from MiSeq were checked for quality using FastQC (http://www.bioinformatics.babraham.ac.uk/projects/fastqc), and low-quality sequences were removed from the ends using FASTX trimmer (http://hannonlab.cshl.edu/fastx_toolkit/). Retained high-quality reads were assembled *de novo* using Velvet 1.2.10 (6) and SPAdes 3.9.0 (7). Assembly statistics were checked by QUAST (8). The contigs were ordered by Abacus 1.3.1 using A. baumannii strain AYE, a multidrug-resistant strain isolated in France (9) (GenBank assembly accession number GCA_000069245), as a reference. Assembled genomes were submitted to NCBI Prokaryotic Genome Annotation Pipeline 4.1 for genome annotation. Assembly statistics and annotation parameters are provided in Table 1. SNPs were detected using BioNumerics 7.6 (Applied Maths, Belgium), relative to the genome of reference strain AYE and one of the strains included in this study (KUSSH36). The SNP analysis showed that five of the six Kuwaiti isolates were genetically close to each other, but all of them were quite distinct from A. baumannii strain AYE (Table 1).

**TABLE 1:** Summary characteristics of whole-genome assemblies of six multidrug-resistant clinical A. baumannii strains isolated in Kuwait

Strain	Mean coverage (×)	*N*_50_ contig size (bp)	No. of contigs	Assembly size (bp)	No. of genes	No. of tRNAs	No. of SNPs (AYE)	No. of SNPs (KUSSH36)	GenBank accession no.
KUSSH13	55	175,365	80	3,887,021	3,811	65	5,327	2,416	NEPY00000000
KUSSH35	52	167,975	60	3,881,261	3,756	63	4,897	2	NEPN00000000
KUSSH36	62	184,821	60	3,882,939	3,758	63	4,899	0	NEPM00000000
KUFAR40	44	141,709	64	3,914,902	3,810	63	4,901	19	NEPJ00000000
KUFAR42	38	138,282	89	3,916,090	3,826	63	4,899	17	NEPH00000000
KUFAR44	64	153,110	69	3,940,599	3,857	62	4,895	11	NEPG00000000

### Accession number(s).

All genome sequences discussed here were submitted to NCBI under BioProject number PRJNA380997 and are available with the accession numbers listed in Table 1.
